# Investigation on failure behavior of collapse column in China’s coal mine based on discontinuous deformation numerical method

**DOI:** 10.1371/journal.pone.0219733

**Published:** 2019-08-06

**Authors:** Jianping Zuo, Zijie Hong, Suping Peng, Yue Shi, Hongqiang Song, Meng Li, Zishan Zhang

**Affiliations:** 1 School of Mechanics and Civil Engineering, China University of Mining and Technology, Beijing, Beijing, China; 2 State Key Laboratory of Coal Resources and Safe Mining, China University of Mining and Technology, Beijing, Beijing, China; University of Science and Technology Beijing, CHINA

## Abstract

Karst collapse column is a serious geological disaster in China’s coal mines. There are various karst collapse columns in coal mine areas, such as Huainan, Huaibei, Xingtai, Lu’an. And they have seriously affected mining safety and geological environment. The present research is focused on analyzing subsidence mechanism and dynamic collapse process. Based on mechanical analysis of thin plate theory, a detailed model of collapse column slipping and bending fracture is constructed to gather the critical conditions of the collapse column roof. The sensitivity parameters analysis shows that both the radius and roof thickness of cave have a great influence on the sliding instability and bending fracture. Meanwhile, the buried depth also affects bending failure. The discontinuous deformation analysis (DDA) method is used to simulate and analyze the collapse process. The numerical results indicate that the stability of inverted funnel collapse column is dominated by the bending stress of roof strata. The movement of columnar collapse column is mainly caused by sliding instability. However, the funnel collapse column is relatively stable, and does not change in the same condition. The displacement field analysis shows that the collapse range of inverted funnel collapse column is obviously larger than that of columnar collapse column, in which its maximum displacement is approximately 1.5 times that of the columnar collapse column. There is no large area collapse on the upper part of the funnel collapse column, and the block system is relatively stable. The principal stress field analysis proves the above results.

## Introduction

There are many collapse columns in mining areas in China. And their morphologies are greatly diverse [1]. Due to their concealed characteristics, the collapse columns become a serious environmental hazard, especially in important mining areas, causing potential damage to human life, property and the environment [2–3]. According to the site data statistics by the end of 2015, there are more than 2800 karst collapses all over 22 provinces in China [4]. Karst column collapses are distributed not only in northern China, but also in southern China. Serious geological and environmental damage has been induced in many coal mines. Some of the affected places are either well developed or densely populated areas, so the damage is serious [5–6].

The collapse columns cause damage not only to the mining industry, but also to railways, highways and karst reservoirs. Therefore, the karst collapse columns have been widely investigated [7–8]. Scholars from America, Russia and Germany are the first to study the karst collapse columns. And some researchers completed the important preliminary analysis of karst collapse [9–10]. At present, the study of collapse columns is featured with constructing theoretical models, and conducting experimental and numerical analysis. Bapulofu develops ‘‘the suffusion effect theory” and explains the mechanism of karst collapse [11]. In the meantime, a lot of other theories are proposed, for example, hydrothermal origin theory [12], thick wall cylinder mechanical model [13], and cyclic expansion karst cave formation theory [14]. In the experimental and numerical analysis, the study of collapse columns is mainly devoted to the cause of formation, distribution of affected areas of collapse columns, pipe erosion and collapse of no-cohesion, and the formation period of collapse columns [15–17].

The existing researches mainly focus on the forming mechanism, water inrush and numerical simulation of the inverted funnel collapse column. However, most of them are based on the theoretical or simplified model. In fact, the collapse mechanism of collapse column can be well studied by numerical calculation. At present, the simulation is mostly based on finite element method or finite difference method, and it is difficult to obtain the deformation field and subsidence mechanisms in the different morphology collapse column. The DDA can simulate the formation mechanism of collapse columns [18–20].

However, one of the most prominent features of DDA in the calculation process is that it needs open and close iterations, that is, repeatedly falling to find the locking position. As a result, DDA is very difficult to complete excavation calculation. Therefore, in this paper, the high efficiency excavation calculation of DDA is realized by changing the calculation method of DDA. In order to analyze the collapse mechanism of different morphologies of collapse columns, this paper establishes a model of collapse column slipping and bending fracture. Meanwhile, based on DDA simulation, the dynamic evolution mechanism of various collapse columns is investigated in detail.

## Morphological analyses of collapse columns

The collapse column is a special morphology of collapse in coal mine fields. Due to the concealed characteristics of collapse columns and their complexity, it is important to understand the existing morphologies and study the failure law. The collapse columns in northern China are mostly distributed in the development zone of karst spring. It is mainly caused by the collapse of overlying strata in the Ordovician karst cave. Some collapse columns show an inverted funnel shape, others present cylindrical morphology and funnel shape. Their horizontal sections are mostly circular or oval [21–22]. Under the direct influence of the Taiyuan Formation limestone water, Huainan and Huaibei mining areas has become the root of the development of the collapse columns. The collapse column is usually shaped like an inverted funnel, and the horizontal section is also round or oval. The collapse column in Shanxi Province is mostly developed at the edge of the basin, and the tectonic movement affects the formation of the collapse column. The overall morphology is similar to that of northern China, few are funnel morphology, and the horizontal sections are mostly round [23–24].

**Fig 1:**
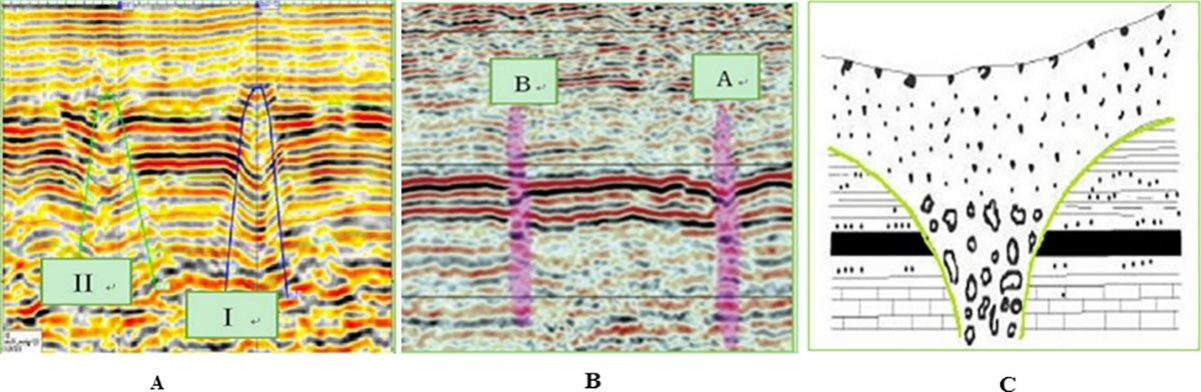
Three typical morphologies of collapse columns. (A) Inverted funnel shaped collapse column ^[25]^. (B) Cylindrical shaped collapse column ^[26]^. (C) Funnel shaped collapse column ^[27]^.

Peng et al. [25] uses the three-dimensional seismic synthesis technique to investigate the distribution and shape of collapse columns in Xieqiao coal mine of Huainan. The seismic section is shown in Fig 1A. The shapes of I and II collapse columns are inverted funnel. Feng et al. [26] uses the morphological index technology to complete the exploration of the coal mine field in the east of Qinnan, Shanxi province, and effectively analyzes the topography of the collapse columns. Both A and B showed similar columnar morphology, as shown in Fig 1B. Zhang et al [27] reveals that most of collapse columns are inverted funnel, columnar and funnel, as shown in Fig 1C. Fig 1A, 1B and 1C are three typical morphologies of collapse columns. This paper will conduct systematic research on these three morphologies of collapse columns.

## Instability model and parameters analysis of collapse columns

The key performance measures mainly include the critical conditions of slip instability and bending failure of collapse columns. In order to analyze the collapse parameters in a more comprehensive way, the cave radius, cave roof thickness and the cave depth are selected as the sensitive parameters to analyze the unstable failure of the collapse column.

### Sliding instability model and sensitivity analysis

**Fig 2:**
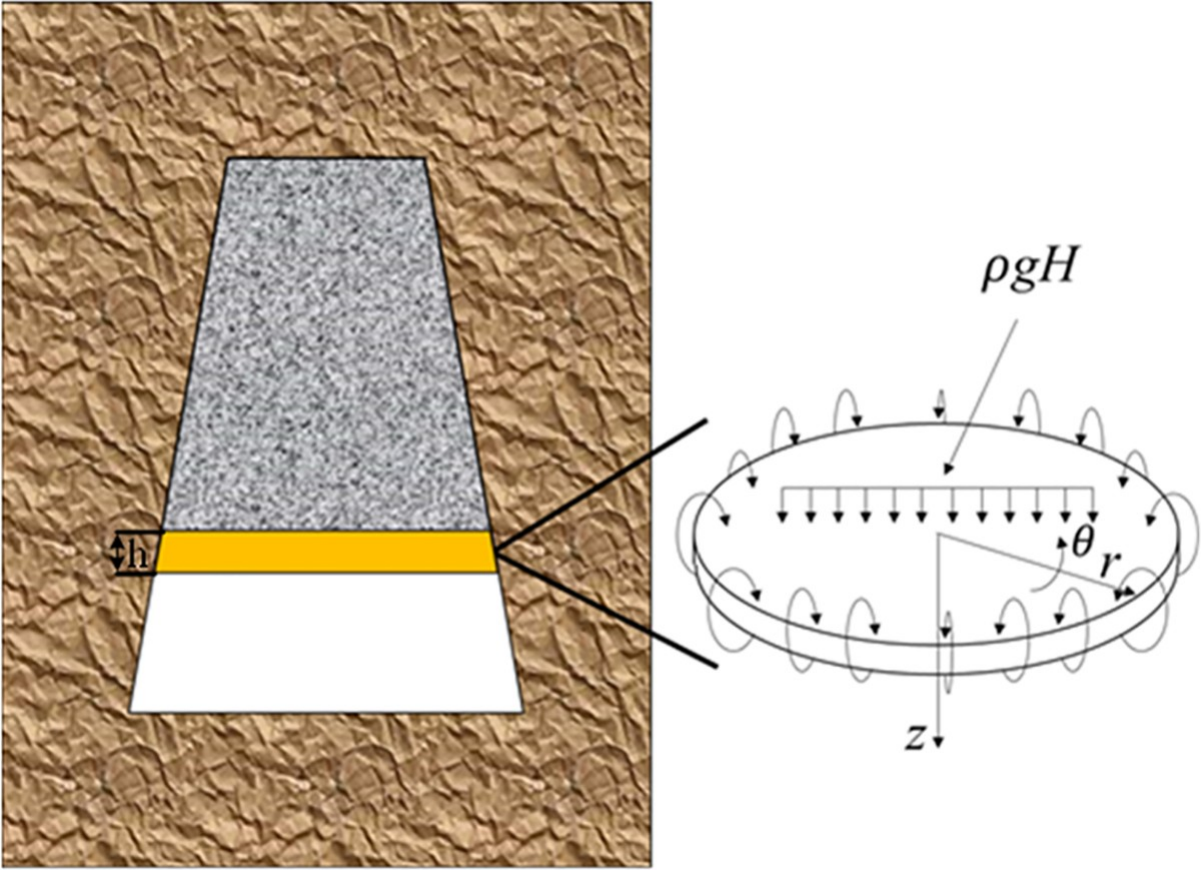
Failure mechanics model of karst cave roof.

After the dissolution of rock mass, the cave roof will be destroyed. Since the horizontal sections of the three morphologies of collapse columns are mostly round or oval, it is assumed that the top plate of the cave is a peripherally fixed thin plate, as shown in Fig 2. γ is the average volume force of the overlying strata, H is the height of the overlying strata.

Vertical stress of karst cave roof [28]:

$$\sigma_{h}=\gamma H$$ (1)

Assuming the lateral pressure coefficient is λ, the horizontal normal stress is:

$$\sigma_{x}=\lambda \gamma H$$ (2)

Assuming that τ is the ultimate vertical shear stress of the boundary element γ_H_ × γ_M_ of the cave roof, according to the Moll-Coulomb criterion:

$$\tau =c+\sigma_{x}\tan\phi$$ (3)

Where c is cohesive force, φ is internal friction angle. From Eqs (2) and (3), the following equation can be obtained [29]:

τ=c+γλHtanϕ (4)

In order to obtain the sliding force around the entire roof model, integral for Eq (4):

$$T=\int_{0}^{L}\int_{H-h}^{H}\left(c+\gamma \lambda H\tan\phi \right)dH.dM$$ (5)

That is:

$$T=\left[ch+\gamma \lambda \tan\phi \left(Hh-h^{2}/2\right)\right]L$$ (6)

The weight of the cave roof and its overlying rock is:

G=γ(H+h)s (7)

Where s = πr^2^ is cross-sectional area, m^2^; h is thin plate thickness, m; L = 2πr is cave circumference, m; r is cave radius, m.

When the load on the karst cave roof exceeds the limit slip force on the whole periphery, the failure will occur:

G>T (8)

The slip instability condition of the collapse column can be obtained:

$$\Delta =\frac{2ch+\lambda \gamma \tan\varphi \left(2Hh-h^{2}\right)}{\gamma \left(H+h\right)r}<1$$ (9)

From Eq (9), it can be concluded that when Δ is less than 1, the cave roof will be destroyed. Thus, the main influence parameters are obtained.

**Fig 3:**
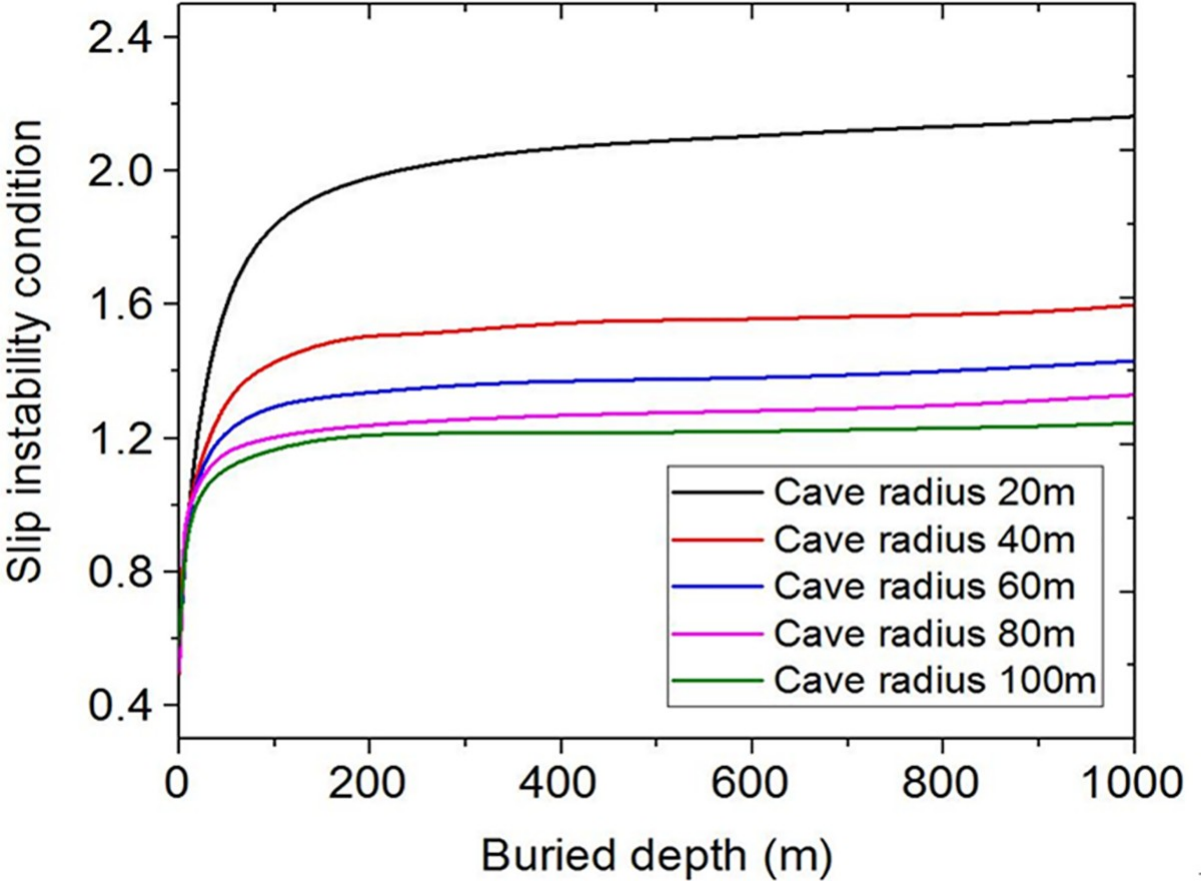
Influence curves of different cave radius.

Taking Xieqiao coal mine as an example. By substituting *H* = 0~1000m, *h* = 20m, *φ* = 30°, *c* = 2.4MPa, *γ* = 25kN/m^3^ into Eq (9), and changing the cave radius, from100m, 80m, 60m, 40m to 20m, the curves of slip instability under different cave radius are obtained (Fig 3). As the cave radius increases, the roof is prone to slip instability. The slip instability condition is greatly affected by the cave radius.

**Fig 4:**
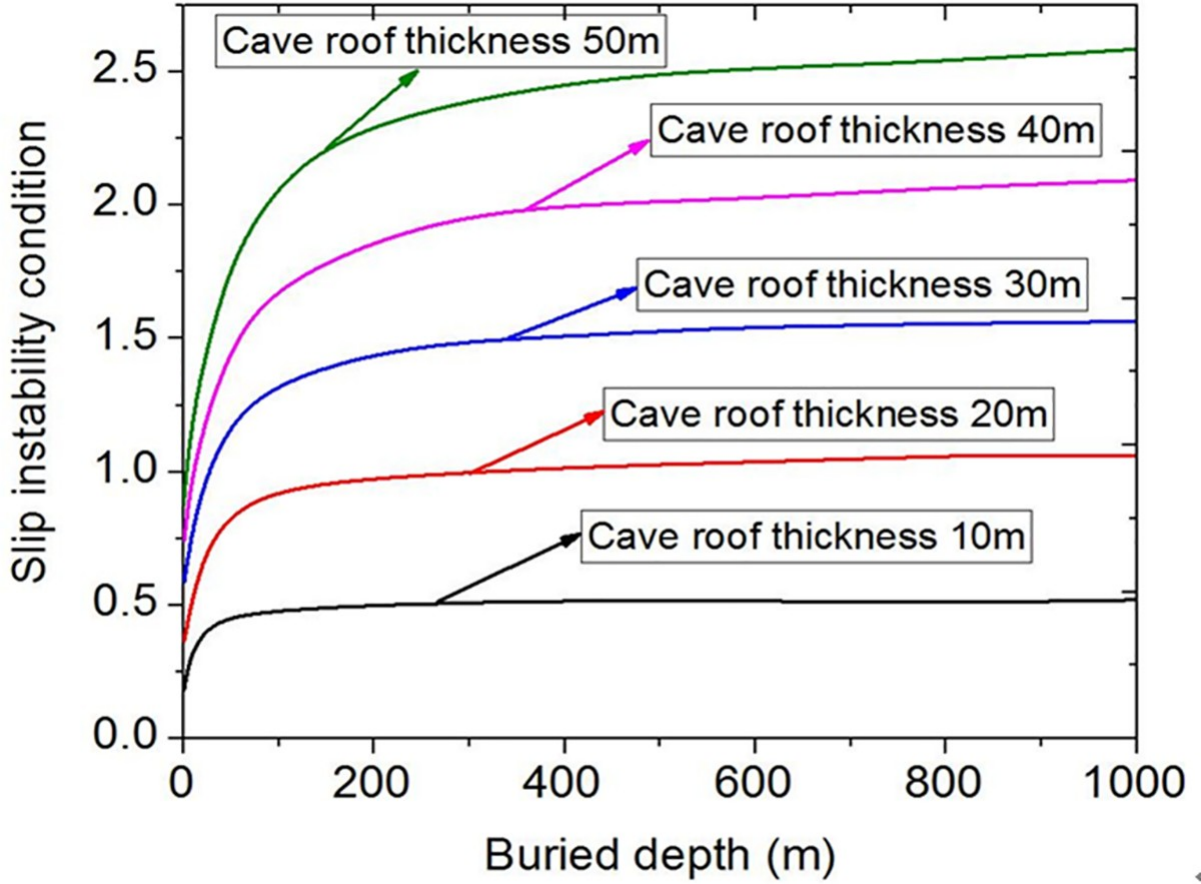
Influence curves of different cave roof thickness.

By substituting *H* = 0~1000m, *r* = 50m, *φ* = 30°, *c* = 2.4MPa, *γ* = 25kN/m^3^ into Eq (9) and changing only the cave roof thickness from 50m, 40m, 30m, 20m to 10m respectively, a set of curves of slip instability are obtained (Fig 4). Fig 4 shows that the slip instability of the roof is very sensitive to the influence of the cave roof thickness. When the thickness interval is 10m, the increase of the slip instability value changes greatly. The smaller the roof thickness is, the easier the slip instability occurs.

**Fig 5:**
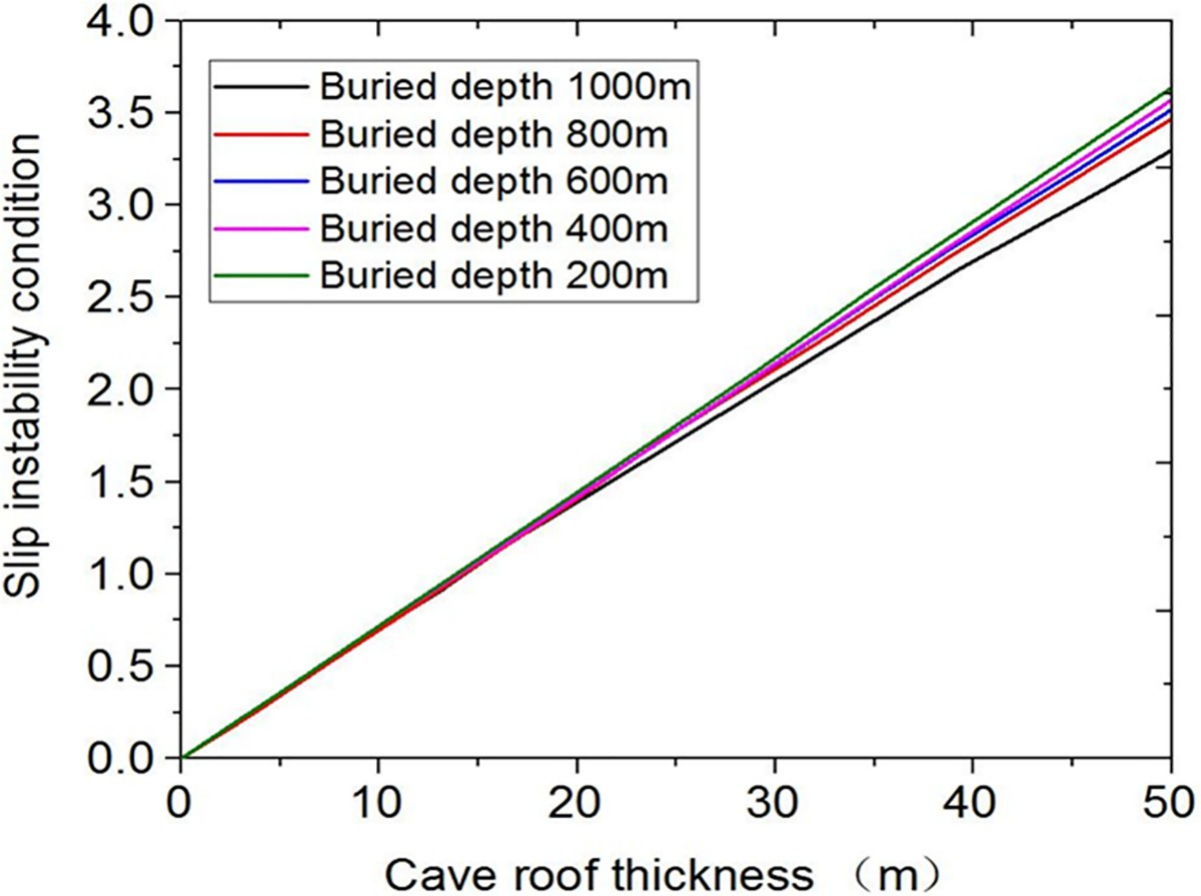
Influence curves of different buried depths.

The influence curves of slip instability conditions under different buried depths are obtained. Fig 5 shows that as the buried depth increases, the slip instability value has not changed significantly. Only when the buried depth is 1000m, the slip instability value shows a slight deviation. Thus the slip instability condition is influenced by two parameters, the cave radius and the cave roof thickness, and the cave roof thickness has larger influence.

### Bending fracture instability model and sensitivity analysis

It is assumed that the karst cave roof is a circular thin plate with peripherally fixed thin plate, when the roof is bend fracture, the condition of instability is solved as follows:

From the polar coordinates [30]:

$$\frac{\partial \rho }{\partial x}=\cos\varphi ,\frac{\partial \rho }{\partial y}=\sin\varphi$$ (10)

$$\frac{\partial \varphi }{\partial x}=-\frac{\sin\varphi }{\rho },\frac{\partial \varphi }{\partial y}=\frac{\cos\varphi }{\rho }$$ (11)

The deflection w is regarded as a function of r and φ, that is w = w (r, φ), and the Eqs (12) and (13) are obtained:

$$\frac{\partial w}{\partial x}=\cos\varphi \frac{\partial w}{\partial \rho }-\frac{\sin\varphi }{\rho }\frac{\partial w}{\partial \varphi }$$ (12)

$$\frac{\partial w}{\partial y}=\sin\varphi \frac{\partial w}{\partial \rho }+\frac{\cos\varphi }{\rho }\frac{\partial w}{\partial \varphi }$$ (13)

$$\mathrm{Where}\;\nabla^{2}w=\frac{\partial^{2}w}{\partial \rho^{2}}+\frac{1}{\rho }\frac{\partial w}{\partial \rho }+\frac{1}{\rho^{2}}\frac{\partial^{2}w}{\partial \varphi^{2}}$$

From the elastic mechanics [30], it is known that the bending differential equations of circular thin plates are:

$$D\nabla^{2}w\left(\frac{\partial^{2}}{\partial \rho^{2}}+\frac{1}{\rho }\frac{\partial }{\partial \rho }+\frac{1}{\rho^{2}}\frac{\partial^{2}}{\partial \varphi^{2}}\right)=\gamma H$$ (14)

Assuming φ is 0, the equations of bending moment and transverse shear force of thin plate in polar coordinates are obtained from elastic mechanics:

$$\mathrm{Where}\;\kappa =\frac{1}{\rho }\frac{\partial w}{\partial \rho }+\frac{1}{\rho^{2}}\frac{\partial^{2}w}{\partial \varphi^{2}}$$

$$\mathrm{That}\;\mathrm{is}:\;M_{\rho }=-D\left(\frac{\partial^{2}w}{\partial \rho^{2}}+\mu \kappa \right)$$ (15)

$$M_{\varphi }=-D\left(\mu \frac{\partial^{2}w}{\partial \rho^{2}}+\kappa \right)$$ (16)

$$\tau_{\rho }=-D\nabla^{2}w\frac{\partial }{\partial \rho }$$ (17)

$$\tau_{\varphi }=-D\nabla^{2}w\frac{1}{\rho }\frac{\partial }{\partial \varphi }$$ (18)

By further transformation, the internal force equation is obtained:

$$\sigma =\frac{12M_{\rho }}{h^{3}}.h_{i};\sigma_{1}=\frac{12M_{\varphi }}{h^{3}}.h_{i}$$ (19)

Because the roof is assumed to be boundary clamped, the upper load is γ H, and the radius is r, thus the boundary conditions can be obtained:

$$w_{\rho =r}=0;{\left(\frac{\partial w}{\partial \rho }\right)}_{\rho =r}=0$$ (20)

The deflection equation is:

$$w\left(R\right)=\frac{\gamma H}{64D}\left(r^{2}-R^{2}\right)$$ (21)

The moment equation is obtained as follows:

$$\begin{array}{l} M_{\rho }=-\frac{\gamma H}{16}\left[\left(3+\mu \right)R^{2}-\left(1+\mu \right)r^{2}\right]\\ M_{\varphi }=-\frac{\gamma H}{16}\left[\left(1+3\mu \right)R^{2}-\left(1+\mu \right)r^{2}\right] \end{array}$$ (22)

When the R is r, the moment is maximum, that is, by substituting *R = r* into Eq (22):

$$M_{\max}=\frac{\gamma Hr^{2}}{8}$$ (23)

The maximum stress occurs at z = h/2, that is:

$$\begin{array}{l} \sigma_{z=h/2}=-\sigma_{z=-h/2}=\frac{6M_{\rho }}{h^{2}}\\ \sigma_{1}{}_{z=h/2}=-\sigma_{1}{}_{z=-h/2}=\frac{6M_{\varphi }}{h^{2}} \end{array}$$ (24)

By substituting Eq (23) into Eq (24):

$$\sigma_{\max}=\frac{3\gamma H}{4h^{2}}.r^{2}$$ (25)

With the neutral axis as the boundary, the upper rock strata is compressed, the lower rock strata is pulled, and the critical condition for the roof damaged is:

$$\sigma_{\max}\geq \sigma_{t}$$

The bending fracture condition of roof is obtained:

$$\Delta_{1}=\frac{4h^{2}\sigma_{t}}{3r^{2}\gamma H}<1$$ (26)

From Eq (26), when the Δ_1_ is less than 1, the karst cave roof is broken. According to the bending fracture instability conditions, the main influence parameters of the collapse column roof are analyzed.

**Fig 6:**
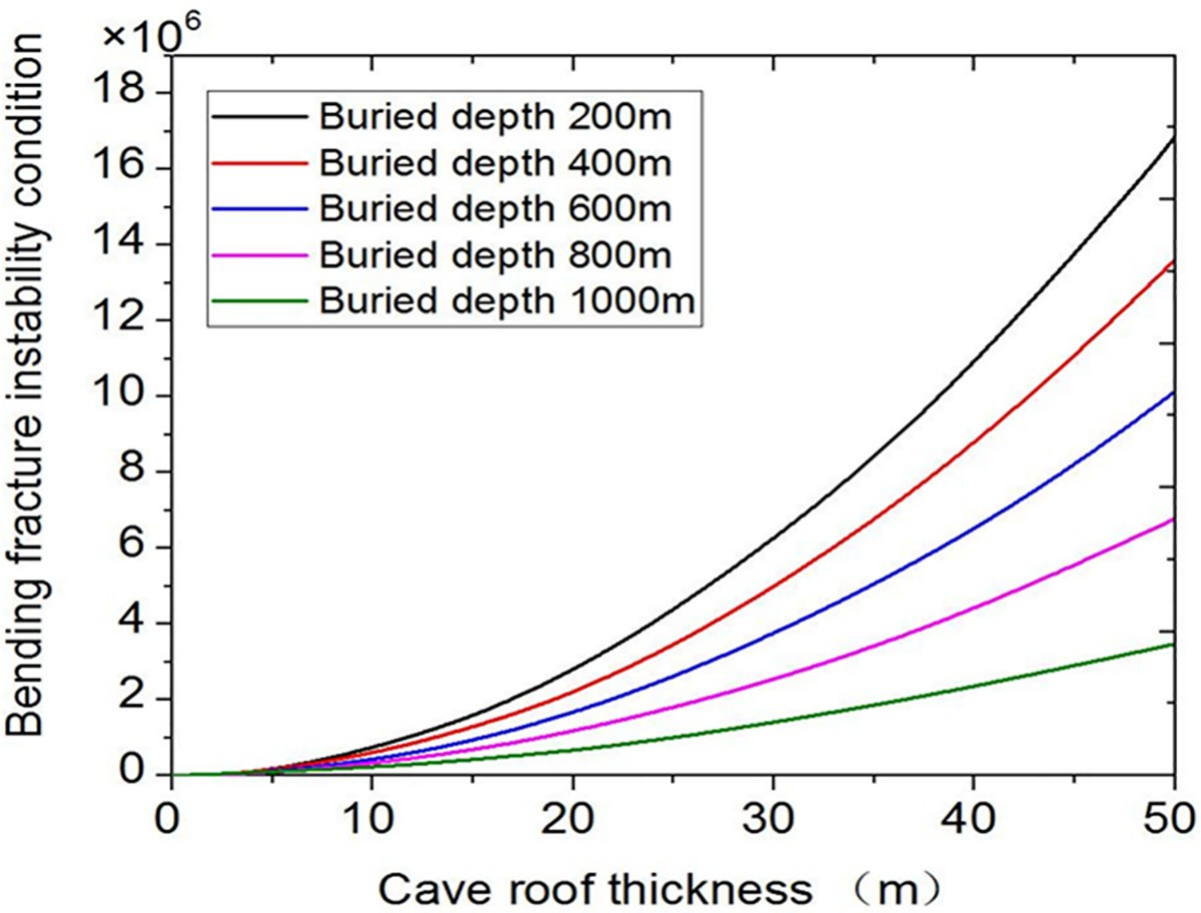
Influence curves of different buried depth.

Only the buried depth is changed from1000m, 800m, 600m, 400m to 200m, respectively, and bending failure effect curves under different buried depths are obtained (Fig 6). Fig 6 shows that as the buried depth increases, the bending failure of roof is easy to occur. Therefore, the bending fracture instability is greatly affected by buried depth.

**Fig 7:**
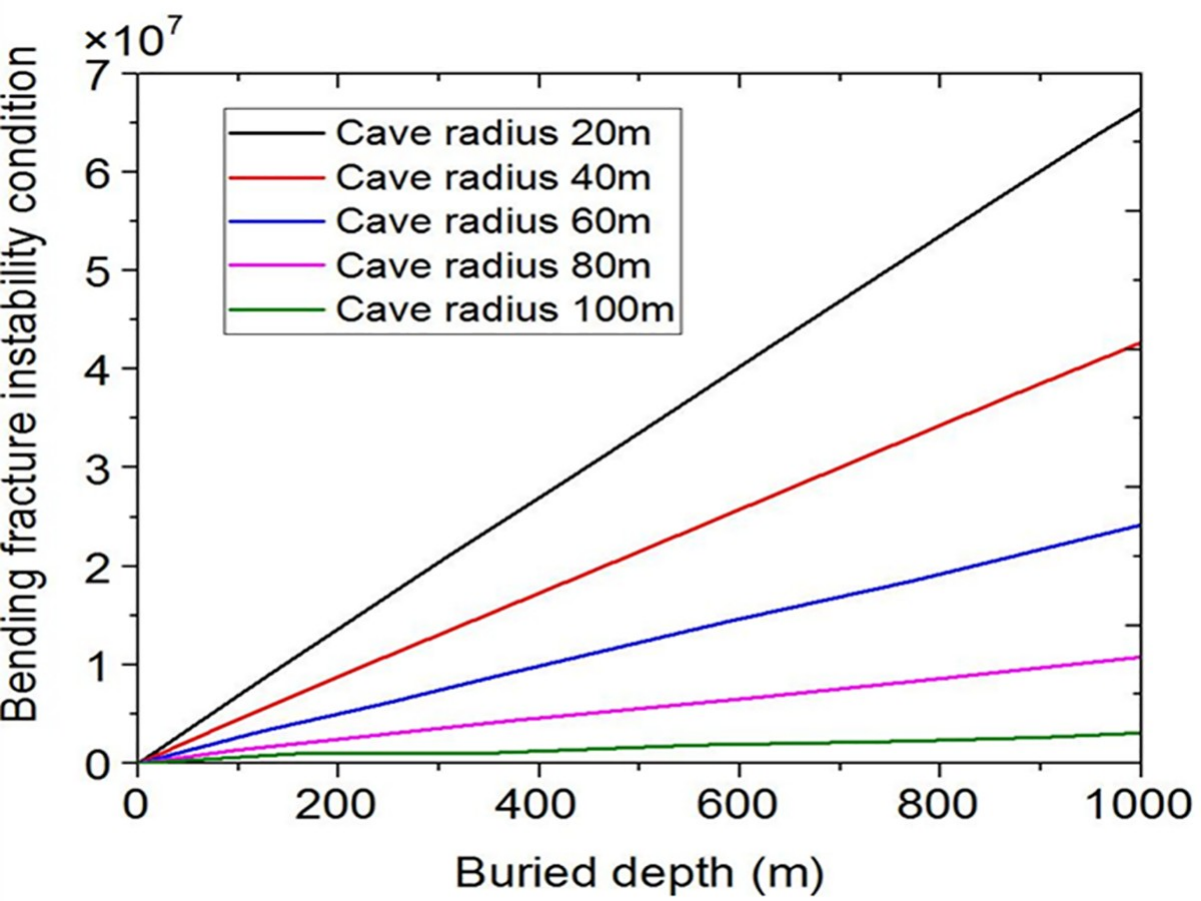
Influence curves of different cave radius.

The cave radius is changed from 100m, 80m, 60m, 40m to 20m, the results are shown in Fig 7. It is clear that the bending fracture instability is greatly affected by the cave radius. The larger the cave radius is, the easier the bending fracture instability occurs.

**Fig 8:**
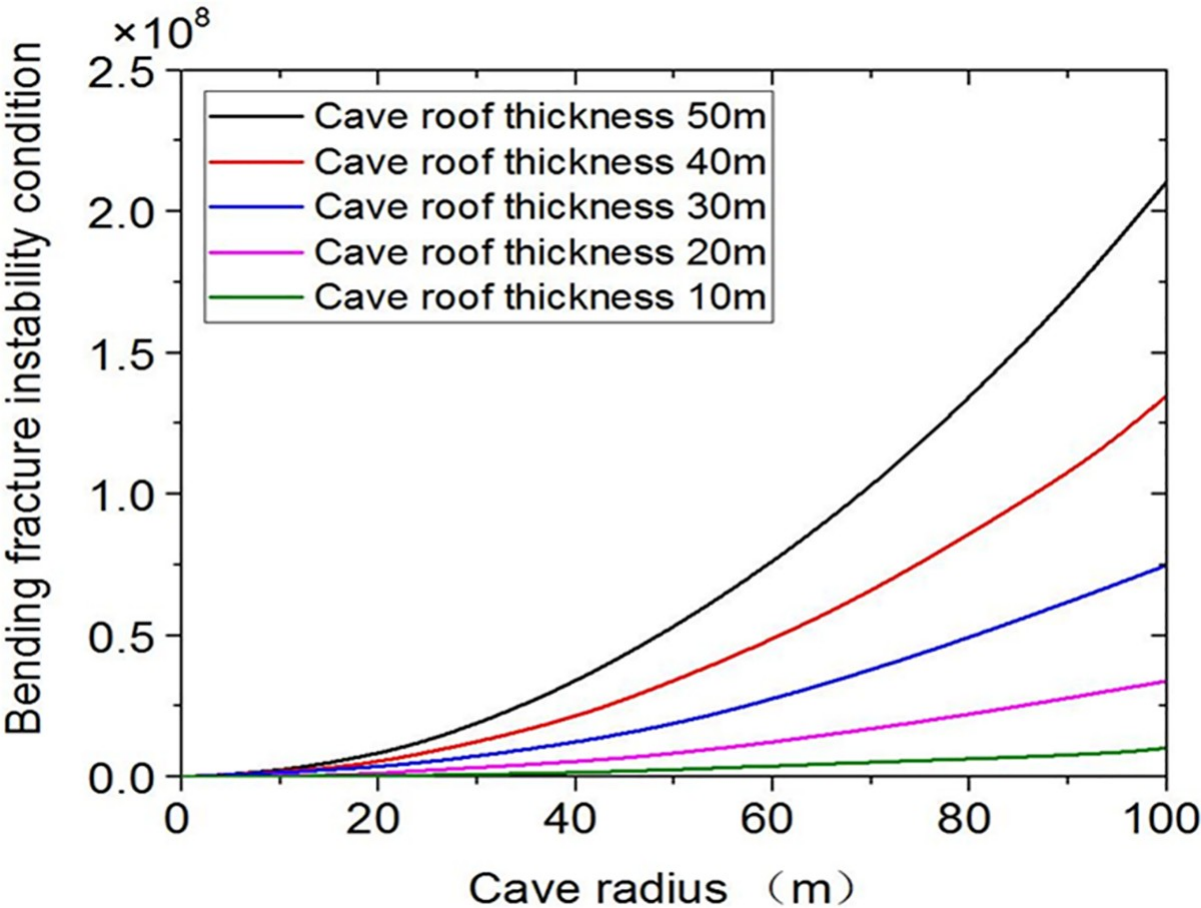
Influence curves of different cave roof thickness.

The influence curves of bending fracture conditions under different cave roof thickness are obtained by changing the parameter of the cave roof thickness (Fig 8). From Fig 8, it is observed that the bending failure instability is more easily affected by the cave roof thickness. Even when the interval is 10m, the impact is still obvious. The above analysis shows that the main sensitive parameters of bending failure instability are different from those of the sliding instability conditions. In addition to the cave radius and the cave roof thickness, the buried depth has significant impact on the bending failure instability.

## Discontinuous deformation analysis (DDA) ^[31]^

The DDA method is characterized by inherent discontinuity, measured displacement, strain or stress as input data. Numerically, the method is to use a set of inequality constraints to minimize the least squares. The input consists of measured data and the output is the block sliding, and joint opening, which are real measures of safety and failure. In the DDA method, rock systems can be regarded as aggregate blocks cut from discontinuous surfaces. The strain of each block is constant, and the deformation of each block is described by the rigid body translation displacement (μ_0_, v_0_) of a specific point (x_0_, y_0_). And the displacement (μ, v) of any point (x, y) is as follows:

$$\left(\begin{array}{c} u\\ v \end{array}\right)=\left(\begin{array}{cccccc} 1 & 0 & -(y-y_{0}) & \left(x-x_{0}\right) & 0 & (y-y_{0})/2\\ 0 & 1 & \left(x-x_{0}\right) & 0 & \left(y-y_{0}\right) & (x-x_{0})/2 \end{array}\right)\left(\begin{array}{c} u_{0}\\ v_{0}\\ r_{0}\\ \varepsilon_{x}\\ \varepsilon_{y}\\ \gamma_{xy} \end{array}\right)$$ (27)

Where *r*_0_ is the rotation angle of the block around point (*x*_0_,*y*_0_), (*ε*_*x*_*ε*_*y*_*γ*_*xy*_) are the strains of the block; i represents the i-th block.

Formula (27) can be given in the following form:

$$\left(\begin{array}{c} u\\ v \end{array}\right)=\left[T_{i}\right]\left[D_{i}\right]$$

The simultaneous equation is obtained by minimizing the square differences or the errors Ψ. The single block is connected to each other by the contact between the blocks and the displacement constraints on the single block and forms a block system. These kinematic requirements and constraints have the form:

$$f(d_{1},d_{2},d_{3},\ldots )=0$$

The penalty term(28) is added to Ψ to impose the kinematic constraints, where *p*>0 is a large positive number:

$$p{\left(f(d_{1},d_{2},d_{3},\ldots )\right)}^{2}=0$$ (28)

Suppose there are n blocks in the defined block system, and the form of the simultaneous equilibrium equation is:

[K][D]=[F]

$$\left(\begin{array}{ccccc} K_{11} & K_{12} & K_{13} & \ldots & K_{1n}\\ K_{21} & K_{22} & K_{23} & \ldots & K_{2n}\\ K_{31} & K_{32} & K_{33} & \ldots & K_{3n}\\ \vdots & \vdots & \vdots & \ddots & \vdots \\ K_{n1} & K_{n2} & K_{n3} & \ldots & K_{nn} \end{array}\right)\left(\begin{array}{c} D_{1}\\ D_{2}\\ D_{3}\\ \vdots \\ D_{n} \end{array}\right)=\left(\begin{array}{c} F_{1}\\ F_{2}\\ F_{3}\\ \vdots \\ F_{n} \end{array}\right)$$ (29)

The i-th row of Eq (29) consists of 6 linear equations

where F_i_ is a 6×1 vector representing the loading on block i. The elements of 6×6 submatrix K_ij_ are determined by:

$${\left(K_{ij}\right)}_{rs}=\frac{\partial^{2}\Psi }{\partial d_{ri}\partial d_{sj}},r,s=1,\ldots ,6$$ (30)

The application of Eq (29) in numerical simulation requires iterative calculation. Deformation and boundary position can be determined by Formula (27). The rigid spring is added or subtracted from the requirement of impermeability and tension at the corresponding contact position. Then Eq (29) is modified and resolved until all contact surfaces meet the requirements.

## Analysis of DDA excavation method

At present, FLAC^3D^ is widely used in analyzing break, so it is compared with DDA in detail as follows:

(a) Comparative analysis of stress field

The overall distribution of the stress field obtained by the DDA simulation is uniform, and the stress concentration is in the local area, the whole distribution of stress field simulated by FLAC is very smooth and uniform. In the whole simulation process, the stress release of the block system calculated by the DDA method is larger due to the large area collapse, so that the overall stress value is reduced and the stress concentration is reduced. During the FLAC simulation, there is only stress release near the roof and floor of the mining face. However, because the calculation unit has not collapsed, the gravity of this part of the element and its own stress aggravates the stress concentration of the deep roof and the two sides of the working face, making the overall analysis too conservative. It shows that the simulation results obtained by DDA method are more accurate.

(b) Comparative analysis of displacement field

The maximum displacement of the DDA method varies greatly, which is consistent with the roof caving characteristics. However, the maximum displacement of FLAC does not change much compared with the displacement of other monitoring points. During the whole calculation process, DDA method give the three-zone distribution of overlying strata caused by face mining, the displacement field is not continuous. FLAC cannot give the distribution of three-zone the calculated displacement field is continuous, and there is no sudden change, the simulation results are quite different from the actual strata collapse.

At present, the state-of-the-art methods to study collapse of collapsed columns are DDA, UDEC and FLAC^3D^. The FLAC and UDEC can solve geotechnical and mining problems, such as analysis of large deformation of soft rock roadway and roof collapse in mining engineering, etc, and the roof failure of collapsed column is the problem of collapse, so we chose the above method for comparison. Although the above method can assume that the object of study is independent block element, these block elements have the shape limitation, their dynamic analysis is not very complete, therefore they cannot solve the structural plane failure problem. DDA can also be used in mining engineering to simulate the roof separation, surrounding rock failure, top coal caving and dynamic response of mining through coal seam, so the DDA method is also selected. However, one of the most prominent characteristics of DDA in the calculation process is that it needs open-closed iteration, that is, repeatedly falling to find the locked position. Therefore, DDA is very difficult to complete the excavation calculation, and still has the advantage of improvement.

In order to realize excavation simulation by two-dimensional DDA, it is necessary to know the whole calculation process. One of the most prominent features of DDA in the calculation process is that it needs open and close iterations, that is, repeatedly falling to find the locking position. As a result, DDA does not have excavation function. To analyze the settlement mechanism of karst collapse column with numerical method, the excavation method of DDA program is put forward. Because of the discreteness of DDA calculation unit, it can also be deleted directly in the calculation process, so as to realize excavation simulation in the true hypothetical sense.

### Realization of DDA excavation technology

The first problem to consider is the judgment of contact. If the calculation unit is suddenly reduced, there should not be an error in the contact judgment. However, because the geometric information of the computing unit is stored in the main program, the geometric information of the unit is an indicator matrix. Therefore, the indicator matrix needs to be modified when deleting the corresponding block. The physical parameters are also stored correspondingly in the program, and the corresponding storage matrix of physical parameters should also be modified. In the last step, the contact displacement is also passed to the time step, the corresponding modification store information and all contacts of the deleted unit will be deleted. The stress matrix, displacement matrix, velocity matrix passed to the time step will also be modified to adjust block number and delete record information to delete block unit. After modification, the program enters contact judgment. Then the contact judgment is finished, the contact transformation is carried out. At this point, the computing unit of the whole program is no longer n, but n-1 or a lot of cuts. At this time step, the main program will do the calculation for the unit that has not been deleted, until the next time it is necessary to delete the unit to make a new change. This process is written as a separate subroutine and added to the front end of the main program, which can complete the deletion of the computational block elements, and realize the excavation simulation.

### Accuracy verification of calculation method

**Fig 9:**
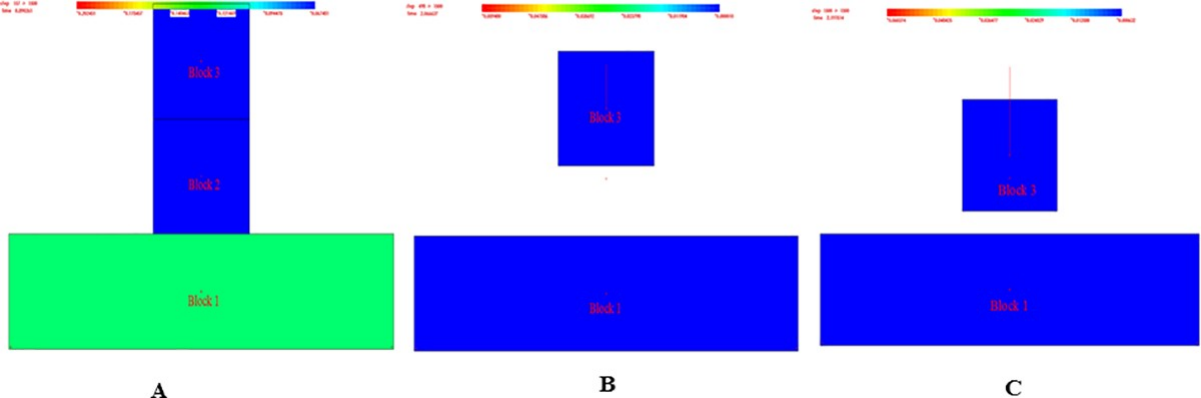
Block calculation process diagram. (A) time step 157, time 0. 89s. (B) time step 498, time 2.06s. (C) time step 1500, time 2.19s.

To verify the accuracy of the proposed method for computing excavation, taking the centroid of three blocks as observation points, the variation of displacement component is analyzed. The stability time of the three blocks is set to 2s, then the block 2 is suddenly removed after 2s. The time removing block is considered 0s, that is, there is no force on the other two blocks in the process of removing the block, and there is no friction to do the work. Then the displacement of block 3 is observed. Using the modified DDA program calculation, the calculation results are depicted in Fig 9.

After the block system is stable, the block 2 is suddenly removed, and block 3 should be in free falling motion. The formula for calculating the displacement of free falling body is as follows:

$$s=\frac{1}{2}gt^{2}$$

**Fig 10:**
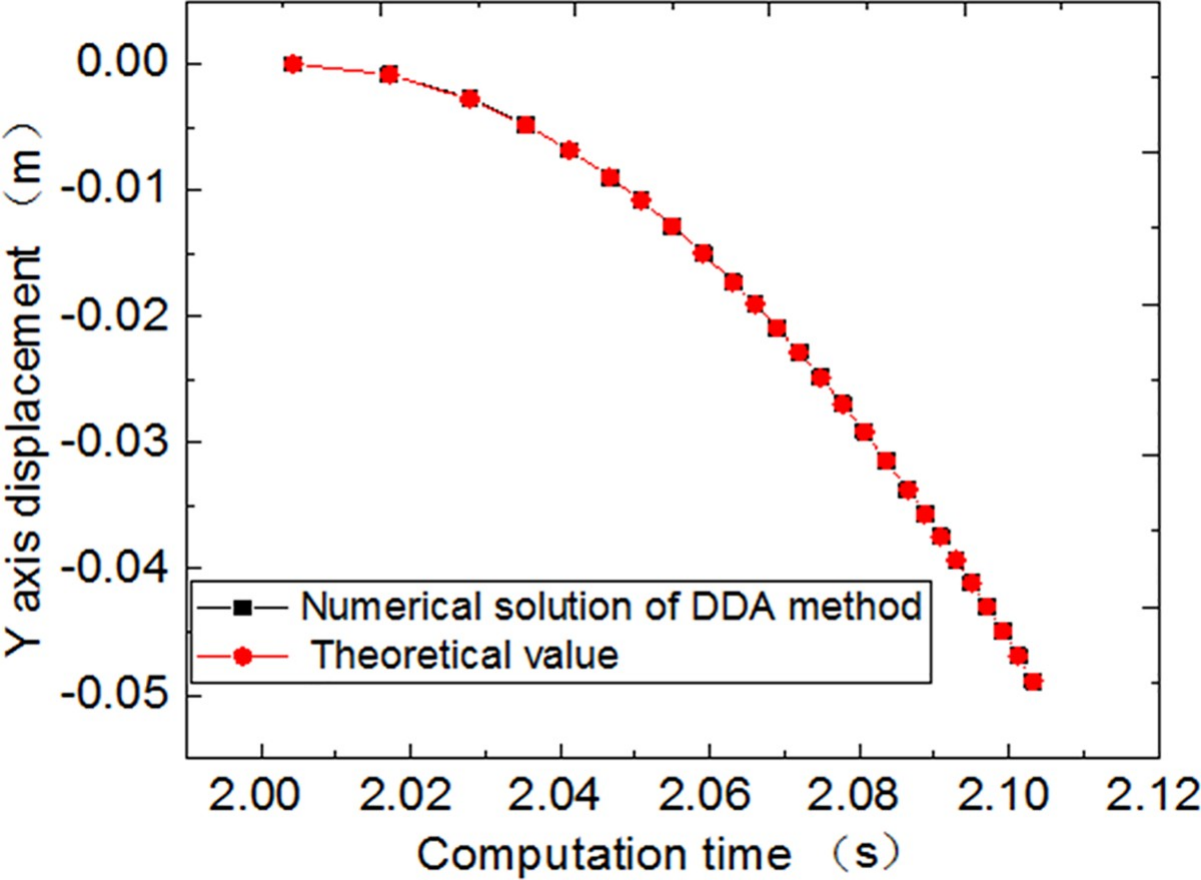
Displacement and time curve.

During the program calculation, the step was 353 steps at the end, and the block 2 was deleted in 2.004s, when 732 steps are calculated, block 3 collides with block 1. The numerical calculation and theoretical calculation of displacement at No.3 observation point are shown in Fig 10.

From the Fig 10, it is observed that the theoretical and numerical solutions of Y-axis displacement of block No.3 are consistent. The rationality of DDA excavation method is further verified.

## Numerical examples

To investigate the subsidence mechanisms and the dynamic collapse process, a discrete numerical method, discontinuous deformation analysis (DDA) is used to study three different morphological collapse columns.

### Simplified model of collapse columns

**Fig 11:**
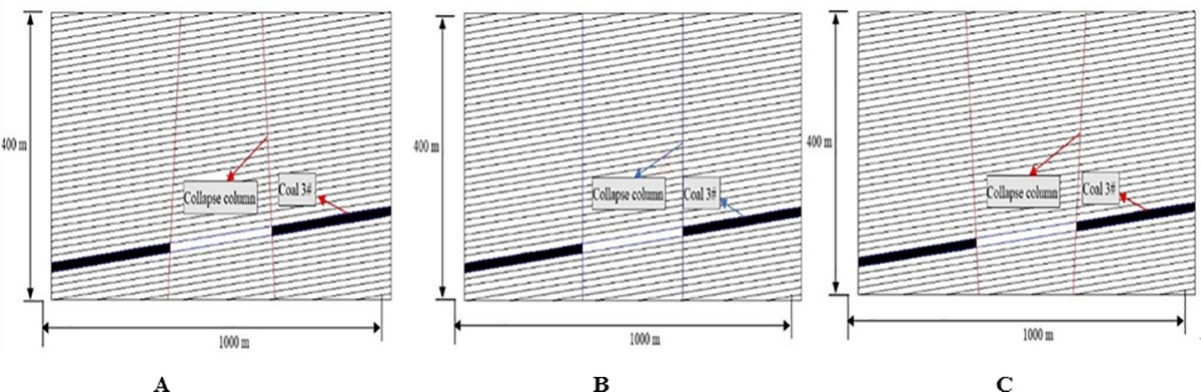
Numerical models of three typical shaped collapse column along mining trend. (A) Inverted funnel shaped. (B) Columnar shaped. (C) Funnel shaped.

A representative area of the Xieqiao coal mine is used as the simulation object and simplified into a two-dimensional plane strain block model along the roadway. The model length is 1000m and the height is 400m. Both the bottom and the side of the model are fixed. In order to simulate the gravity of overlying strata, the top surface of the model is approximately uniformly distributed, and the collapse column diagram is shown in Fig 11.

According to the geological data, the average dip angle of rock joint surface is 4°, so the random joint inclination angle is 86°, the average spacing of joint is 11m, and the average length is 200m. Except for the different morphologies of the collapse columns, the model establishment parameters and calculation parameters are the same, and the model hollowing length is designed to be 150m. The mechanical parameters of rock strata is shown in Tables 1 and 2.

**Table 1:** Mechanical parameters outside the collapse columns.

Coal and rock	Unit weight (g/cm^3^)	Elastic modulus (GPa)	Poisson's ratio	Friction angle (°)	Cohesive strength (MPa)
** Epipedon**	1.80	28.00	0.298	20.00	2.00
**Mudstone**	2.65	32.62	0.297	36.00	4.00
**Middle-grained sandstone**	2.65	79.52	0.298	36.00	11.00
**Coal #3**	1.44	2.80	0.463	31.81	2.09

**Table 2:** Mechanical parameters of the collapse columns.

Coal and rock	Unit weight (g/cm^3^)	Elastic modulus (GPa)	Poisson's ratio	Friction angle (°)	Cohesive strength (MPa)
** Epipedon**	1.70	14.00	0.30	20.00	1.00
**Mudstone**	2.54	16.31	0.30	36.00	2.00
**Middle-grained sandstone**	2.54	39.76	0.30	36.00	5.00
**Coal #3**	1.22	1.40	0.463	31.81	1.04

### Dynamic collapse process of collapse columns

**Fig 12:**
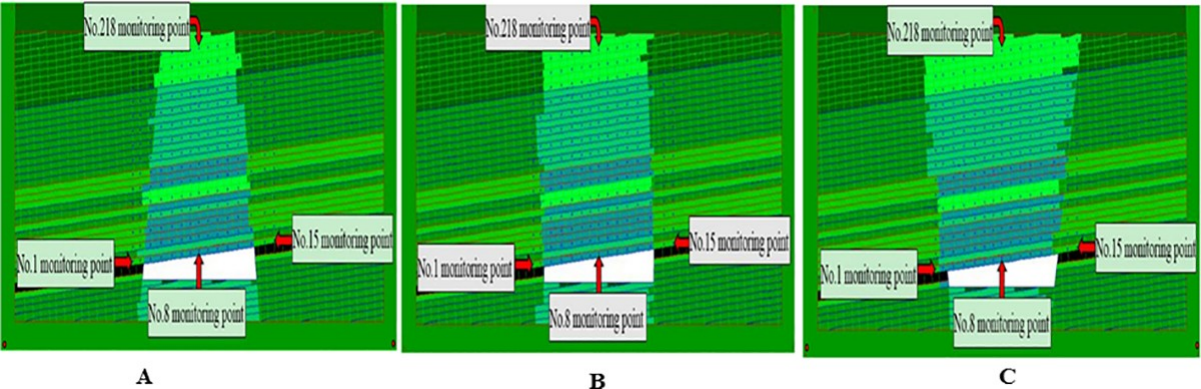
Numerical model of three typical collapse column. (A) Inverted funnel. (B) Columnar. (C) Funnel.

The collapse column model found in coal mines is shown in Fig 12, in which different colors distinguish different attributes of materials. The empty space in the model is the empty area excavated. In order to observe the movement and stress state of the block in detail, the model sets up the multi-layer omnidirectional observation point, that is, the blue circle with the uniform distribution of fifteen strata. The number of observation points increases from left to right and from bottom to top. DDA can not only study the motion and stress state of blocks in different regions, but also analyze the distribution of the three zones. The red circle in the bottom is a fixed point mark. The two fault dip angles are 72° in Fig 12A, and the friction angle is set as 30° of fault parameters, without cohesive strength. In Fig 12B and 12C, the two fault dip angles are 90° and 70°, respectively.

**Fig 13:**
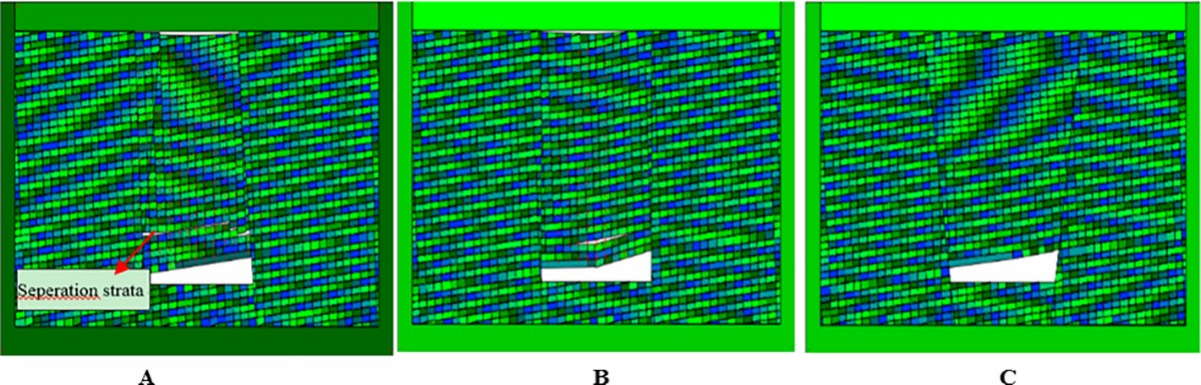
Simulated operation at 2400 time step. (A) Inverted funnel. (B) Columnar. (C) Funnel.

**Fig 14:**
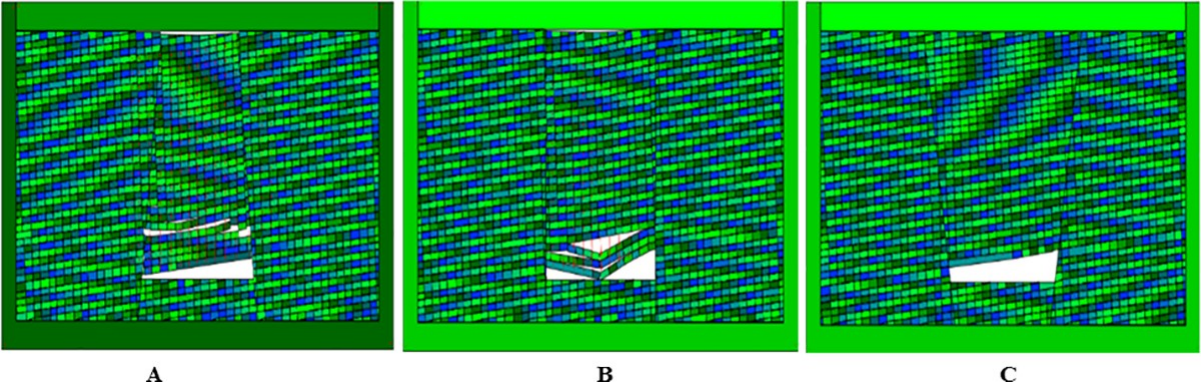
Simulated operation at 3200 time step. (A) Inverted funnel. (B) Columnar. (C) Funnel.

**Fig 15:**
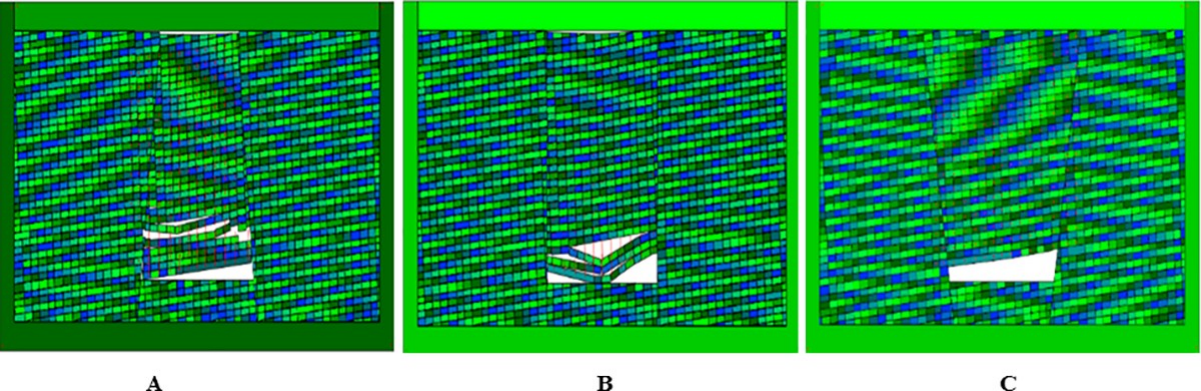
Simulated operation at 4000 time step. (A) Inverted funnel. (B) Columnar. (C) Funnel.

The calculation time step is set to 4000 steps, allowing maximum deformation rate of 0.1%. The simulation results are shown in Figs 13, 14 and 15. It is observed that before the inverted funnel collapse column appears separation strata, the immediate roof of the hollow area controls the load of the overlying strata, and functions as the “key stratum”. Therefore, the deformation of the overlying strata is relatively small, and the internal structure of the collapse column basically does not change. However, due to the erosion of groundwater, the rock strata is decompressed, and the load of the overlying strata is transferred to the strata in the hollow area and outside of the collapse column. As the dissolution effect continues to increase, a large number of the collapse column rocks are taken away, forming a large stress field. A significant stress concentration effect is observed, and it further leads to the occurrence of the separation strata (Fig 13A). Once the separation strata occurs inside the collapse column, the bearing structure loses its bearing capacity rapidly under the action of corrosion, and the collapse begins to occur. Meanwhile the upper strata becomes load-bearing carrier, and bending deformation occurs. At this stage, the separation strata continues to occur and gradually increases, resulting in large bending deformation of the overlying strata and accelerating subsidence (Fig 14A). When a quarter of the rock above the cave is destroyed, the energy accumulated in the overlying rock stara may be released instantly. It will lead the rock stara to give way and eventually form a collapse column (Fig 15A).

The internal structure of the columnar collapse column does not change much before it becomes the separation strata. As the dissolution time approaches, the karst caves continue to expand, resulting in stress concentration effects, accompanied by separation strata. When the columnar collapse column is separated, the immediate roof of the cave column loses its supporting capacity by long-term erosion. While the overlying rock mass rupture degree increases, the stability of adjacent strata gradually decreases. The roof of the karst cave is also affected by the internal joints and faults. The rock mass is prone to collapse in the direction of easy sliding. With the sharp increase of roof load, the instability failure will occur when the load of the cave roof exceeds the maximum sliding force around the surrounding rock (Fig 13B and Fig 14B). At the same time, the semi-elliptical stress field is formed above the collapse column, which exacerbates the occurrence of layer separation and eventually collapses, as shown in Fig 15B.

When the funnel collapse column is in the same operation step, there is no separation strata. The internal structure of the collapse column does not change, and there is no large-scale collapse deformation from beginning to end. Only the rock mass in the upper part of the cavity has small displacement deformation, and basically no deformation occurs, as shown in Fig 13C, Fig 14C and Fig 15C.

### Displacement field analysis of collapse columns

**Fig 16:**
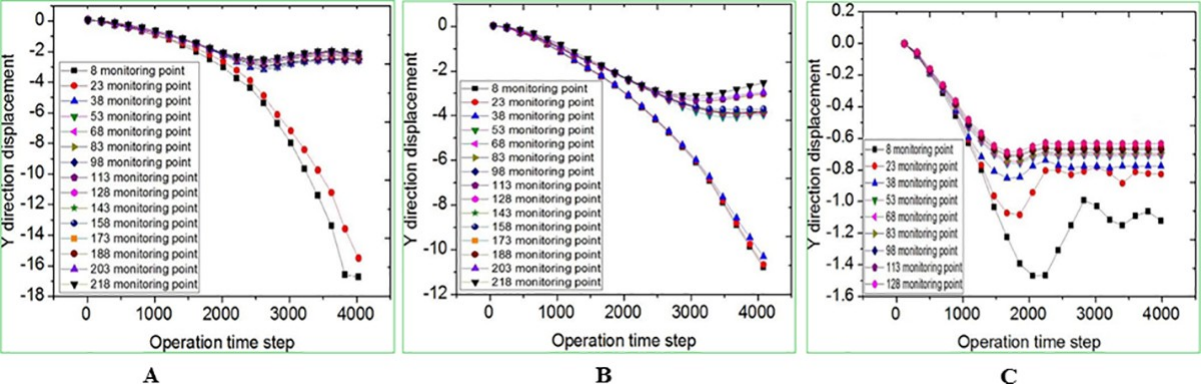
Displacement curve of monitoring point on the same X-axis in the Y-axis direction. (A) Inverted funnel. (B) Columnar. (C) Funnel.

**Fig 17:**
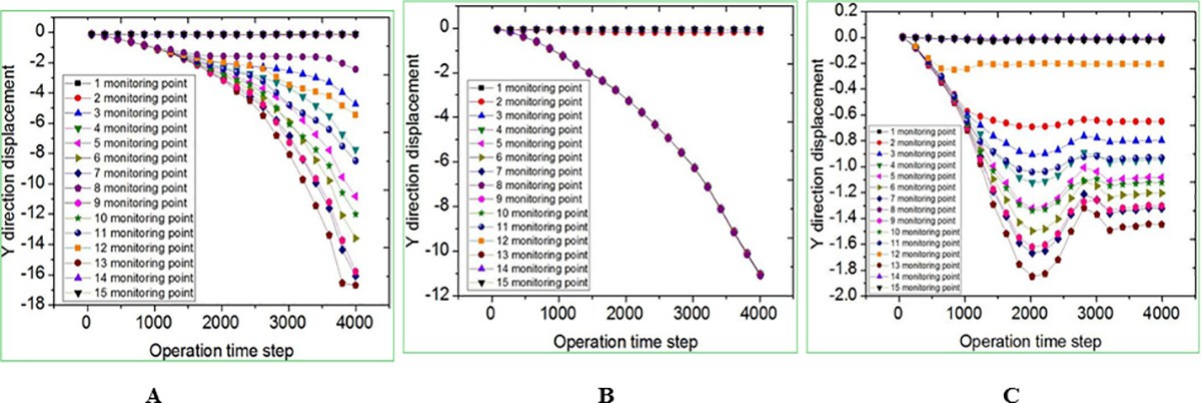
Displacement curve of monitoring point No.1-15 in the direction of Y-axis. (A) Inverted funnel. (B) Columnar. (C) Funnel.

The displacement field results are shown in Figs 16 and 17. It can be seen from Fig 16A that only 2 monitoring points (No.8, No.23) suffer significant collapse, which is the closest to the top of the cave. As the monitoring distance increases, the collapse decreases gradually. Besides, the collapse values of the other monitoring points are basically the same, and the maximum collapse value is only 3mm. Due to the arching effect, the overlying rock tends to be stable after large deformation. Fig 17A shows the displacement curve of the bottom row of monitoring points in the y axis. It is known that except for a few monitoring points (No.1, No.2, No.15) outside the karst cave, there is no collapse; and the rest blocks show the collapse, with a maximum value of 17mm.

According to the above analysis, the collapse of the inverted funnel collapse column shows the following characteristics: the collapse column mass is affected by fault space position to have a large area of collapse. The instability collapse block basically occurs in the collapse column, and few occurs near the collapse column. The left side of the collapse column block is more unstable than the right side, because of the influence of 86 degree random joints. At the same time, the overall collapse shows a tendency to tilt to the left.

As shown in Fig 16B and Fig 17B, there are three monitoring points where the collapse column has large collapse, that is, No.8, No.23 and No.38. The large displacement and deformation also occur in all blocks above the location of the No.23 monitoring point. And the overlying rock mass tends to be stable after large deformation due to the action of arch (Fig 16B). Only two monitoring points of funnel collapse column, No.8 and No.23, have relatively large collapse, but only 1.2mm and 1.5mm. All the other blocks have no displacement change, and the overlying strata tends to be stable (Fig 16C). In the same operation, the maximum subsidence value of inverted funnel collapse columns and columnar collapse columns is 11 times and 7 times greater than that of funnel collapse columns. And the minimum subsidence value of both is larger than the maximum value of funnel collapse columns.

From the displacement curve of the bottom row of monitoring points in the direction of the y axis, it can be known that the inverted funnel collapse column has a large degree of collapse with the exception of a few monitoring points outside the cavity. The maximum subsidence value of inverted funnel collapse columns and columnar collapse columns is 9.5 times and 6.0 times greater than that of funnel collapse columns (Fig 17B). The displacement of funnel collapse column in the center of the cavity is relatively large. Under this condition, there is no large-scale collapse of the rock mass in the upper part of the cavity. The subsidence value is obviously small (Fig 17C. The above analysis further verifies the stability of the three morphology.

### Principal stress field analysis

**Fig 18:**
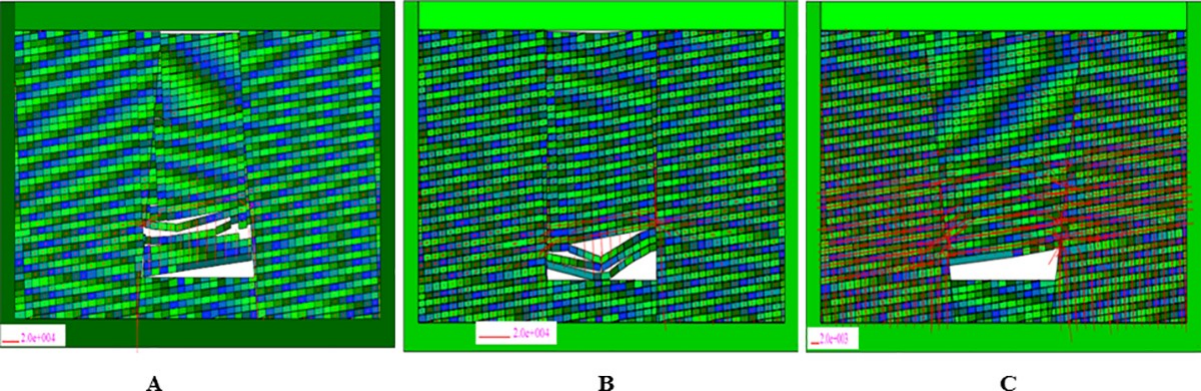
The final principal stress of different collapse columns. (A) Inverted funnel. (B) Columnar. (C) Funnel.

The principal stress line of the block in the model is shown in Fig 18, the length of the red line indicates the magnitude of the principal stress. From the Fig 18A, it can be seen that the stress concentration at the left end of the cavity bottom is obviously higher than that at the right. Besides an arched stress ring is formed above the caving zone, the upper rock block does not fall down, and the block begins to stabilize. In addition, stress concentration areas also appear in the upper strata of the cavity, which are located on the left and right side of the collapse column, respectively. As shown in Fig 18B, it is observed that the stress concentration at the left and right end of the cavity is very obvious. The collapse of the caving zone is similar to that of the inverted funnel. An arched stress ring is also formed above the caving zone, and the block starts to stabilize. This conclusion of stability can be proved by Fig 17B. As can be seen in Fig 18C, the stress concentration zone mainly occurs at the left and right ends of the top of karst cave, the overall stress distribution is relatively uniform. And the stress release of the block in the collapse column is more obvious. The rock blocks near the top of the cavity are mainly subjected to horizontal pressure. Therefore, they are stabilized in the block system without falling down.

## Discussions

### Discussion on mechanism of slip instability

At the initial stage of collapse, the immediate roof above the karst cave can be regarded as the key stratum, and the blocks are interlocked with each other, which weakens the instability and deformation ability between the blocks. With the extension of dissolution, the karst cave becomes larger, the degree of rupture of the overlying rock mass increases, but the stability of the collapse column decreases. At the same time, due to the influence of spatial joints, rock mass collapse occurs in a direction conducive to sliding. When the collapse column fault presents a central axis symmetry, the roof arch collapse position appears in the center of the roof arch. During the failure stage of the collapse column, the energy caused by stress is suddenly released, and it leads instantaneous collapse and causes the formation of collapse columns. When the load on the roof of the cave exceeds the limit slip force on the whole periphery, that is, Δ<1, it will be destroyed. The simulation results of the columnar indicate that the final collapse failure degree is small, and the collapse failure process is similar to slip failure.

### Discussion on mechanism of bending fracture instability

In the initial stage of the collapse column, the immediate roof plays a supporting role due to the existence of the direct roof of the inner hollow area. Therefore, the top of karst cave is in a stable state. As the erosion of groundwater increases, the collapse column rocks are gradually washed away. Then the stress concentration is formed, which accelerates the occurrence of the separation strata. The collapse occurs inside the collapse column, and the upper strata presents bending deformation. When the roof of the karst cave reaches the critical failure condition, that is, *σ*_max_≥*σ*_*t*_, the roof will be broken, presenting a bending deformation. As shown in Figs 12A, 13A and 14A, when the overlying strata on the inverted funnel collapse column appears the phenomenon of separation strata, it will result in large bending deformation of the upper strata and accelerate the settlement. Once the inverted funnel collapse column encounters a high stress area, it will release its accumulated energy and cause rock collapse. The roof fall accident in Xieqiao Coal Mine happens by this mechanism.

The failure law is different due to different morphologies of collapse columns. The reasons are listed as follows: the columnar collapse column is similar to the cylinder; in the geological tectonic movement, the stress concentration area is reduced. Compared with the inverted funnel collapse column, the collapse height decreases, the collapse value is obviously less than the inverted funnel and the pressure is weakened. The stress concentration distribution area and stress value are weaker than those of the inverted funnel collapse column.

Although the morphology of the funnel collapse column is irregular, its damage is the least. The possible reason is that the stress of this form of collapse columns is approximately parallel to the stress direction of tectonic stress or that the angle is small, which is least influenced by the tectonic stress. The stability of the block system is the greatest.

## Conclusions

In the article, different morphologies of collapse columns are investigated. The collapse model of collapse columns is established, and the dynamic collapse process of collapse column with different morphologies is simulated by DDA. The main conclusions are obtained as follows:

(1) Based on the existing detection results, the collapse columns are classified into three morphologies: inverted funnel, columnar and funnel. The critical conditions of sliding instability and bending fracture of the collapse column roof are established by analyzing the model of the collapse column.

(2) Combined with the slip and bending failure instability conditions, the parameter sensitivity analysis was carried out. The main sensitive parameters of bending fracture instability are different from those of slip instability conditions. Besides the cave radius and the cave roof thickness, the parameters which have a significant influence on the instability are also affected by the buried depth.

(3) The discontinuous deformation analysis (DDA) results indicate that the collapse process of the inverted funnel is mainly caused by bending fracture; the columnar collapse column mainly shows slip instability; and the stability of funnel collapse column is the best.

(4) The displacement field analysis shows that: the subsidence displacement of inverted funnel is the largest. The monitoring subsidence displacement is more than 1.5 times that of the columnar, and its stability is the worst. The cave upper of funnel does not show subsidence, and the upper strata forms an arch structure, so the whole tends to be stable. The above results prove the rationality of DDA computing method.
